# Editorial: New insights into intracellular pathways and therapeutic targets in CNS diseases

**DOI:** 10.3389/fncel.2025.1559821

**Published:** 2025-02-04

**Authors:** Lisa Gherardini

**Affiliations:** Institute of Clinical Physiology, Consiglio Nazionale delle Ricerche (CNR), Siena, Italy

**Keywords:** glioblastoma (GBM), Alzheimer's, epilepsy, neurodegeneration, microenvironment, drug repurposing, local delivery

The future of therapy in the realm of central nervous system (CNS) disorders unquestionably lies in the development of targeted, personalized treatments. The ever-expanding toolkit of cutting-edge technologies is enabling researchers to gain unprecedented insights into the complexities of the human brain, revealing intricate physiological pathways that govern neurological health. This editorial introduces a selection of studies featured in this Research Topic of *Frontiers in Cellular Neuroscience*, all of which focus on the dysregulation of intracellular signaling networks that play a critical role in neurological diseases. These studies offer not only fresh perspectives on the pathophysiology of these conditions but also new hope for developing effective treatments and, potentially, cures.

At the heart of these innovations is the recognition of the brain's unique and highly specialized nature. Every structure, every neuronal circuit, and each individual cell in the brain holds a specific role in regulating emotions, memory, cognition, and behavior. The fine-tuned balance of these elements must be preserved for healthy neurological functions. This delicate balance poses a critical question for the development of new therapies: what are the consequences of suboptimal treatment, particularly in diseases that affect the brain? How can the risks and benefits of experimental therapies be carefully weighed to ensure that patient dignity is respected, particularly when the stakes are so high? (Maidment et al., 2024).

In this Research Topic, we present a selection of studies that help to cope with these complex questions. One such contribution comes from Marino et al., who explore the phenomenon of “Brain Fog,” a cognitive condition often experienced by patients undergoing radiotherapy for brain cancer. Though the term may initially seem metaphorical, Marino et al. argue that “Brain Fog” is, in fact, a distinct post-treatment condition that can have long-lasting effects on cognitive function. This phenomenon is particularly pronounced in glioblastoma patients, who are frequently subjected to radiotherapy. The study underscores the critical need to understand the biological mechanisms behind Brain Fog, particularly the role of protein misfolding and microglial activation in neurodegeneration. Marino et al.'s work highlights the importance of studying the brain's microenvironment—a rapidly growing area in neuroscience that holds tremendous potential for the development of personalized, patient-centered treatments. Understanding how the brain's immune cells, such as microglia, interact with tumor cells and surrounding tissues could lead to more effective therapeutic strategies, reducing the cognitive side effects that often accompany cancer treatments.

However, treating glioblastoma remains one of the most formidable challenges in modern medicine. Glioblastoma is an aggressive form of brain cancer with a particularly poor prognosis and limited treatment options. Despite some advancements in surgical techniques and radiation therapy, survival rates for glioblastoma patients have remained low, with few meaningful breakthroughs since the approval of Temozolomide in the late 1990s (Jezierzański et al., 2024). The tumor's intracranial location, combined with the notorious very limited permeability of the blood-brain barrier, presents unique obstacles to drug delivery (Fukushima and de Groot, 2024). Moreover, the inherent heterogeneity of glioblastoma cells means that, even if one aspect of the tumor is targeted effectively, other subpopulations may remain unaffected, leading to tumor recurrence. Chiariello et al. delve into this problem, exploring novel approaches to intracranial drug delivery. They review a range of techniques that aim to bypass the blood-brain barrier and facilitate the targeted delivery of therapeutic agents directly to the tumor site. Despite early promise, many of these methods have encountered challenges, and the quest for more effective drug delivery platforms remains an ongoing endeavor. Chiariello et al.'s work emphasizes the need for continued innovation, particularly in the development of “smart materials” that can respond to the unique characteristics of the tumor microenvironment (Nehra et al., 2021). These advanced drug delivery systems may 1 day offer more precise, localized therapies that could significantly improve the prognosis for glioblastoma patients.

Another promising avenue of therapeutic development lies in drug repurposing—an approach that allows researchers to investigate existing, FDA-approved drugs for new therapeutic indications (Rani et al., 2024). This strategy offers several advantages, including shorter development timelines, lower costs, and reduced risks associated with safety and toxicity. One example of this approach is the work of Pallavicini et al., who investigate the potential of the citron kinase inhibitor Lestaurtinib in the treatment of medulloblastoma, a common and aggressive pediatric brain tumor. Lestaurtinib, which was originally developed for its effects on the Sonic Hedgehog (Shh) signaling pathway, exhibits a poly-pharmacological profile, including the ability to induce DNA damage and promote apoptosis. Pallavicini et al.'s study suggests that Lestaurtinib may hold promise as a repurposed treatment for medulloblastoma, highlighting the therapeutic potential of rethinking old drugs for new applications. This research exemplifies the power of drug repurposing in accelerating the availability of novel treatments for brain tumors and other CNS diseases.

Beyond traditional pharmacological approaches, this Research Topic also explores the role of endogenous molecules in CNS diseases. As the brain sciences continue to evolve, even well-known molecules are revealing new and unexpected functions. A fascinating example is the work by Cavalu et al., who investigate the role of orexin, a neuropeptide involved in regulating arousal, sleep-wake cycles, and appetite. Their research uncovers a dual role for orexin in both promoting neurodegeneration and potentially preventing cancer, depending on the context. While orexin may contribute to the survival of cancer cells, its involvement in apoptosis regulation in the brain suggests that it may also have a neuroprotective role under certain conditions. This discovery opens the door to further research into orexin-based therapies, potentially targeting orexin receptors to either alleviate neurodegenerative diseases or limit cancer cell proliferation.

Similarly, Shaikh et al. examine the therapeutic potential of modulating dopamine transporters in the context of Alzheimer's disease. Dopamine, a neurotransmitter essential for regulating mood, attention, and motor function, is often dysregulated in neurodegenerative diseases, including Alzheimer's (Saggu et al., 2024). By targeting dopamine transporters, Shaikh et al.'s work suggests that it may be possible to modulate dopamine signaling in a way that alleviates both motor and cognitive deficits seen in Alzheimer's patients. The ability to fine-tune dopamine signaling represents an exciting area of research with the potential to offer new treatment options for Alzheimer's and related disorders.

Microglia, the resident immune cells of the central nervous system, have also emerged as central players in the pathology of many neurological diseases (Abellanas et al., 2025). Once thought to be passive supporter of neuronal health, microglia is now recognized for its active role in regulating neuroinflammation and neuronal plasticity. Recent research has uncovered significant regional heterogeneity in microglial phenotypes, suggesting that microglia exhibit distinct behaviors depending on the brain region and disease context. In this Research Topic, several studies explore regional variations and the potential therapeutic implications of modulating microglial function. For example, research by Smith et al. reveals that microglia in neurogenic regions appear to be in a “primed” state, with heightened immune responses and antigen presentation capabilities, which could influence both their role in neurogenesis and their contribution to the pathogenesis of epilepsy.

One particularly exciting development is the discovery of post-translational modifications of microglial proteins, such as lactylation, which may influence microglial behavior and contribute to disease progression. By targeting specific post-translational pathways including histone acetyltransferases (HATs) and histone deacetylases (HDACs), researchers may be able to tune microglial hyperexcitability, either enhancing their protective functions or suppressing their harmful effects (Chen and Zhu, 2024). This underscores the importance of considering the precise role of microglia in different areas of the brain when designing therapies aimed at modulating glial functions that could offer new therapeutic opportunities for treating conditions such as Alzheimer's and CNS prion disease.

Therefore, the identification of activation markers and modulators that influence microglial function opens potential avenues for precision medicine. By targeting specific microglial subtypes or activation states, it may be possible to tailor treatments to individual patients, maximizing efficacy while minimizing side effects. Personalized approaches therefore suggest a promising future for the treatment of CNS diseases, where therapies are not one-size-fits-all but instead are designed to address the unique characteristics of each patient's disease.

In conclusion, studies highlighted in this Research Topic offer a glimpse into the future of CNS disease treatment. Through the exploration of novel intracellular pathways, the reimagining of existing therapies, and the identification of new therapeutic targets, these studies pave the way for more effective, personalized interventions. As our understanding of the molecular and cellular underpinnings of neurological diseases continues to deepen, we are better equipped to develop treatments that not only address symptoms but also target the root causes of these conditions. The journey toward personalized and precision medicine offers hope for a future where CNS diseases are no longer an insurmountable challenge, but treatable conditions with well-defined and effective therapeutic solutions.
